# High-Speed Energy-Efficient Resonant Modulator on a Graphene-Organic Hybrid Platform

**DOI:** 10.1021/acsphotonics.6c00793

**Published:** 2026-10-24

**Authors:** Xinzhi Zhang, Ping Ma, Tobias Blatter, Yannik Horst, Wolfgang Heni, Alexandros Emboras, Tatsuhiko Watanabe, Nikolaus Flöry, Lukas Novotny, Delwin L. Elder, Larry R. Dalton, Juerg Leuthold

**Affiliations:** † 31060 ETH Zurich, Institute of Electromagnetic Fields (IEF), Zurich 8092, Switzerland; ‡ University of Science and Technology, School of Microelectronics, Hefei, Anhui 230023, P. R. China; § Polariton Technologies, Adliswil 8134, Switzerland; ∥ ETH Zurich, Integrated Systems Laboratory, Zurich 8092, Switzerland; ⊥ 27219 ETH Zurich, Photonics Laboratory, Zurich 8093, Switzerland; # 7284 University of Washington, Department of Chemistry, Seattle, Washington 98195-1700, United States

**Keywords:** graphene, nonlinear organics, electro-optic modulator, resonant modulator, silicon photonics, optical communications

## Abstract

Resonant electro-optic modulators are of interest for encoding electrical signals to the optical domain with an option to perform simultaneous wavelength-division multiplexing while reducing the modulation energy consumption. Here, we demonstrate a compact, high-speed, and energy-efficient resonant modulator exploiting the ultrafast characteristics of graphene and organic materials, enabling efficient electro-optic modulation on the silicon platform. Graphene, with its extraordinary electronic and optical properties, is used as a transparent electrode material that brings the radio frequency field close to the photonic mode with minimal extra losses, while the organic material provides a high linear electro-optic coefficient. This arrangement provides both efficient light modulation in a compact device with an active length of 50 μm and a large modulation bandwidth of ∼35 GHz. High-speed data transmission experiments demonstrate a line rate of 320 Gbit/s, while a rate of 128 Gbit/s is achieved under CMOS-compatible voltages with energy consumption of ∼10.1 femtojoules per bit. The presented technology offers a practical and promising route toward integration of graphene into high-speed communication links.

## Graphical abstract

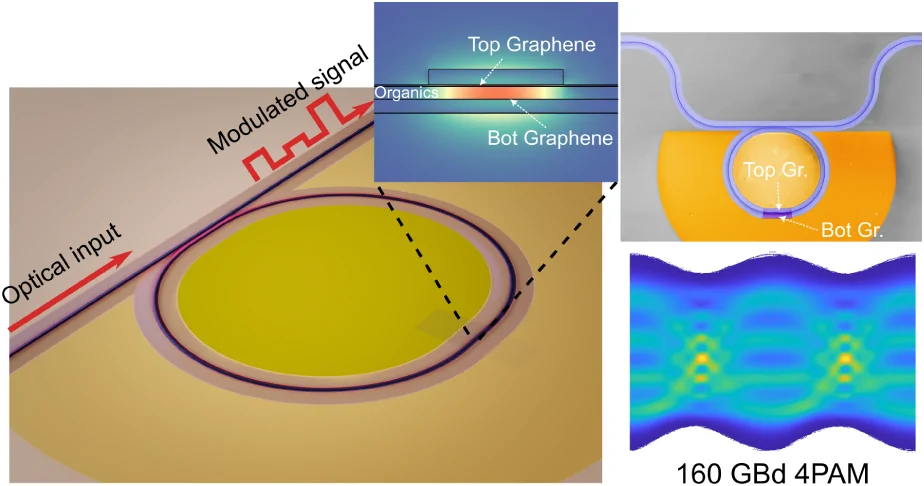

## Introduction

The exponential growth of global data traffic in the long-reach communications,^1^ along with the rapid increase of computational capacity for artificial intelligence in the short-reach scenarios^2^ have drastically intensified the demand for optical interconnects capable of high-speed and energy-efficient operations.^3^ Resonant electro-optic modulators are critical in this regard, offering compact footprints and compatibility with intensity modulation/direct detection (IM/DD) architectures. Their capability to support both modulation and wavelength-division multiplexing (WDM) further boosts the possible data capacity.^4^ The sharp resonance enhancement enables direct driving by CMOS voltages without further amplification, and thus minimizes the energy consumption.^5^

Various platforms have been investigated to realize resonant modulators that meet the key requirements of high modulation efficiency, large bandwidth, low energy consumption and compact footprint. Silicon microring modulators (Si MRMs) have been widely employed in industry due to their compactness and CMOS compatibility.^6–8^ However, silicon as an active medium shows weak nonlinearities based on free-carrier plasma dispersion,^9^ limiting the modulation efficiency. Additionally, the ultrahigh quality factors (*Q*-factors) of silicon resonant cavities impose photon-lifetime limitations,^10,11^ constraining the electro-optic bandwidth as a result of the inherently narrow filter linewidth. This constraint comes on top of other bandwidth-limiting factors, such as the circuital resistor-capacitor (RC) time constant and limitation imposed by carriers’ drift and diffusion processes. The ultrahigh *Q*-factors also impose the need for strict thermal control.^12^ Thin-film lithium niobate (TFLN) ring modulators utilizing Pockels effect have also demonstrated large *Q*-factors that limit their bandwidth,^13^ and their relatively weak mode confinement and field overlap often necessitate millimeter- to centimeter-scale footprints. Conversely, plasmonic-organic hybrid (POH) electro-optic modulators have been developed for ultrahigh speed optical communications.^14–16^ They offer extremely compact footprints, ultralow RC time constants^16,17^ as well as strong light-matter interactions with the highly nonlinear organic (NLO) materials.^18–20^ For plasmonic resonant modulators, the *Q*-factor is reduced by the optical losses from the metallic structures, and therefore it no longer limits the bandwidth of resonant modulators.^21–24^ Such modulators have successfully achieved high speed and great temperature stabilities.^23^ As an alternative to the plasmonic electrodes made by bulk metals, one might consider two-dimensional (2D) materials. 2D-materials, in particular graphene,^25–28^ have emerged as promising candidates for optoelectronic devices owing to their exceptional optoelectronic and mechanical properties. Employing the carrier-based nonlinearities, graphene-based resonant electro-absorption modulators (EAMs)^29^ achieve intensity modulation by shifting the Fermi level and therefore modulation of the roundtrip losses. However, their high-speed performance is often limited by slow capacitive charging. Energy efficiency of the EAMs is also compromised due to the large device capacitance. Furthermore, operation at an optimal Fermi level typically requires electrical doping via a large DC bias. To address these challenges, hybrid integration of graphene with NLO materials offers a promising approach by combining superior electronic properties of graphene with low optical losses and efficient modulation.

In this work, we present a novel graphene-organic hybrid (GOH) resonant electro-optic modulator that leverages the low optical losses and high conductivity of graphene electrodes, the strong nonlinearity of functional NLO materials, and the reduced energy consumption from the resonant enhancement of a racetrack resonator. The fabricated prototyping device features a compact footprint of 65 μm × 75 μm. A 3-dB bandwidth of ∼35 GHz was measured. The highest line rate of 320 Gbit/s was achieved in back-to-back IM/DD data transmission measurements. Furthermore, with a CMOS-compatible voltage of only 0.4 V_p_, 128 Gbit/s signal was successfully transmitted featuring a low energy consumption of ∼10.1 fJ/bit.

## Device Concept

The proposed modulator comprises a racetrack resonator evanescently coupled with the bus waveguide, see Figure 1(a). In the racetrack resonator, two graphene electrodes sandwich a thin layer of NLO material along the active phase shifter section shown in Figure 1(b).^30^ The layer stack of the active device is depicted in Figure 1(c). A transverse magnetic (TM)-polarized optical mode is confined by a bottom silicon slab and a top amorphous silicon (a-Si) strip, with most of the optical power inside the NLO material. The cross-sectional view of the waveguide and optical fields of the supported TM mode are plotted in Figure 2(a) and Figure 2(b). By applying a radio frequency (RF) electrical signal in the GHz range on the graphene electrodes, as shown in Figure 2(c), a strong electric field in the order of MV/cm is established across the NLO layer, and the phase of the passing optical carrier is modulated by means of the Pockels effect.^31^ Therefore, intensity modulation is achieved at the output as a result of the induced resonance shift.

**Figure 1:**
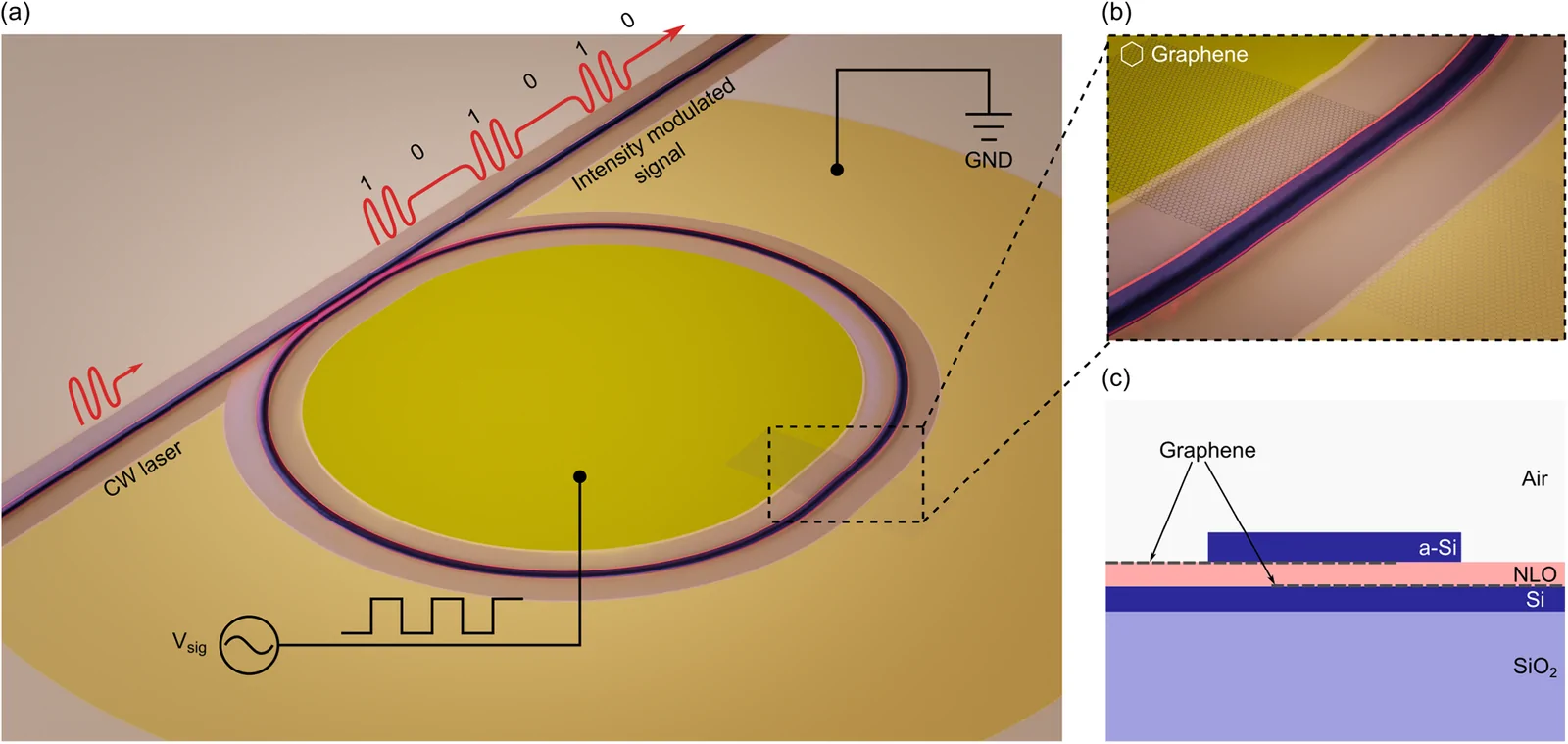
Concept of a graphene-organic hybrid (GOH) resonant modulator. (a) Artistic render of the GOH resonant modulator. By applying a signal voltage to the contact pads, as well as to the graphene electrodes, intensity modulation of the continuous-wave (CW) laser carrier is achieved at the output. (b) Zoom-in render of the active phase shifter embedded in the racetrack resonator. (c) Layer stack of the embedded active phase shifter. The waveguide is constructed by a bottom silicon slab, a top amorphous silicon (a-Si) strip and in between a layer of nonlinear organic (NLO) material sandwiched by two layers of graphene electrodes, creating a strong light-matter interaction inside the NLO material.

**Figure 2:**
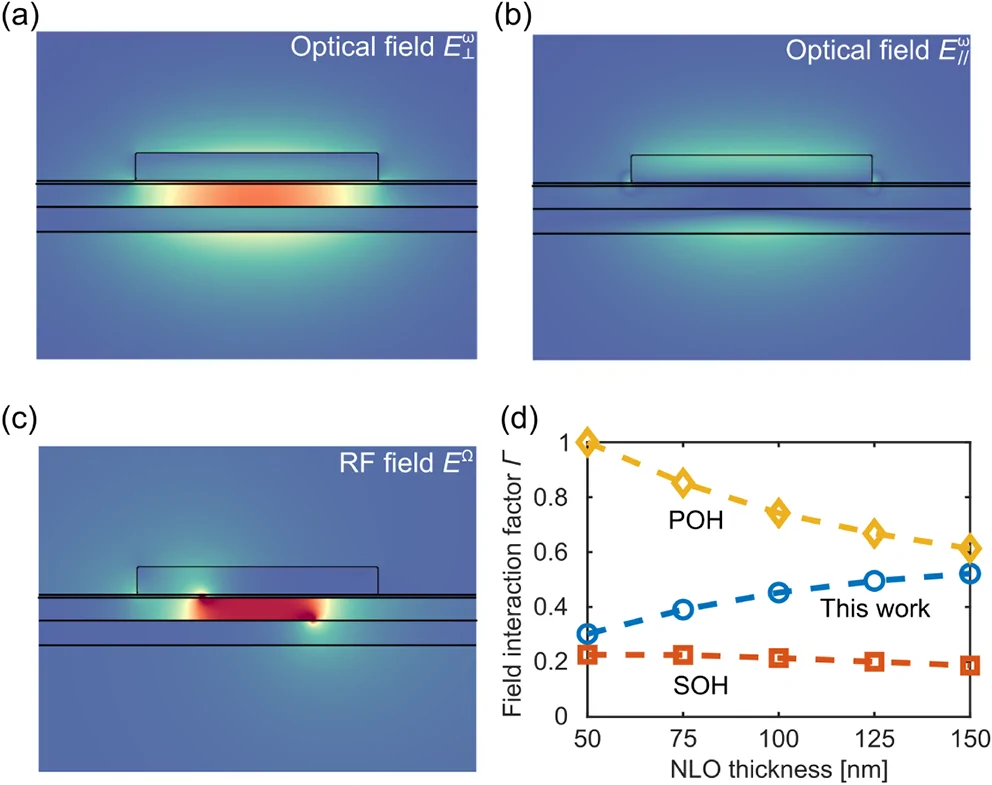
(a) Magnitude of out-of-plane *E*_⊥_ component of the TM-polarized photonic mode. The major electric field component normal to graphene reduces the propagation losses induced by graphene absorption. (b) Magnitude of in-plane *E*_||_ component of the photonic mode, which is minimized by device’s geometry optimization. (c) Magnitude of the driving electric field of the applied RF signal. The large overlap between the electrical and optical fields enhances the light-matter interaction. resulting in high modulation efficiency. (d) Field-interaction-factors of this work compared with those of the plasmonic-organic hybrid (POH)^14,18^ and silicon-organic hybrid (SOH)^34,35^ platforms for a varying NLO thickness. With a 100 nm thick NLO layer, the field-interaction-factor is calculated to be ∼0.5 in the GOH configuration.

The GOH resonant modulator is designed for high modulation efficiency and a compact footprint. Comparing the guided optical field in Figure 2(a) with the RF field in Figure 2(c), a strong spatial overlap between the mode profiles is observed, ensuring a strong interaction between the RF and optical fields. The field interaction factor,^32,33^ that indicates the modulation efficiency for a field-effect electro-optic modulator, is calculated to be ∼0.5. As a comparison, simulations show that this value exceeds the interaction factors obtainable from silicon-organic hybrid (SOH) electro-optic modulators,^34,35^ as plotted in Figure 2(d). When properly activated, the employed NLO material provides an ultrahigh Pockels coefficient,^19^ which, in combination with the strong field interaction, ensures a high modulation efficiency within a short device length. The highly efficient GOH phase shifter is embedded in a racetrack resonator with a radius of 30 μm, resulting in a compact footprint of 65 μm × 75 μm. The device is designed with a free spectral range (FSR) of ∼4 nm, enabling the device for WDM applications. Time-domain small-signal analyses show that the racetrack resonator enhances the optical modulation amplitude (OMA) by over 60% compared to a Mach–Zehnder modulator of the same active length under push–pull operation (see Supporting Information 3). The high modulation efficiency allows a low driving voltage and a compact device design to further enhance the energy efficiency and integration density.

The proposed device design promises both a low insertion loss and a large bandwidth. The atomically thin graphene leads to an extremely small overlap between the electrode material and the optical fields, reducing the propagation losses. Moreover, as a result of graphene’s anisotropic optical conductivity,^36,37^ it exhibits negligible absorption for out-of-plane optical fields and non-negligible absorption for the field components parallel to the graphene plane. Accordingly, our device operates with TM-polarized photonic mode that does not hold a dominant field component parallel to the graphene plane, as depicted in Figure 2(a) and Figure 2(b). The device geometry was optimized to further suppress the optical in-plane electric fields to minimize the propagation losses (see Supporting Information 1). Simulations predict an optical propagation loss as low as 0.004 dB/μm for monolayer graphene electrodes. Besides, the atomic thinness of graphene electrodes eliminates the coupling losses from the passive access waveguide to the active phase shifter. The low propagation loss and minimized coupling losses lead to a moderate *Q*-factor that enhances the OMA through optical resonance, while avoiding the inherent RF bandwidth constraints of ultrahigh-Q resonators.^38^ The electro-optic bandwidth is mainly determined by the device’s RC time constant. Graphene’s extraordinary electronic properties such as low resistivities and high carrier mobilities contribute to a small resistance of the device. As for the device’s capacitance, the NLO material sandwiched by graphene electrodes possesses a low relative permittivity (<10),^35^ and a layer thickness of approximately 100 nm ensures a small device capacitance in the range of femtofarad. Furthermore, the high modulation efficiency enables a shortened active section, thereby further reducing the overall capacitance of the device. The low resistance and capacitance of the device contribute to a large RC-limited bandwidth.

In conclusion, the design of the GOH resonant modulator takes advantage of both graphene electrodes and the highly nonlinear NLO material so that it allows us to compress the strong RF field inside the functional material for efficient modulation without bringing in excessive optical losses. The design also features a racetrack resonator that further improves the modulation performance by optical resonance while keeping a compact footprint. It also promises high-speed operations from the low RC constant and a moderately short photon lifetime.

## Results

### Fabrication and Characterization

The proposed devices were fabricated on a silicon-on-insulator wafer with a 110 nm thick silicon device layer. The false-color SEM image of a fabricated device is shown in Figure 3(a). The bus waveguide is connected to two grating couplers (GCs) for laser feeding in and out of the device. Optical power is coupled to the racetrack resonator via the directional coupler formed by the closely placed bus and racetrack waveguides. In the resonator, graphene electrode sheets are inserted in a section of the waveguide to sandwich the NLO material (JRD1),^39,40^ making up the active phase shifter. The top and bottom graphene electrodes extend to the metal pads inside and outside of the racetrack, respectively, to provide electrical contacts for microwave probes.

**Figure 3:**
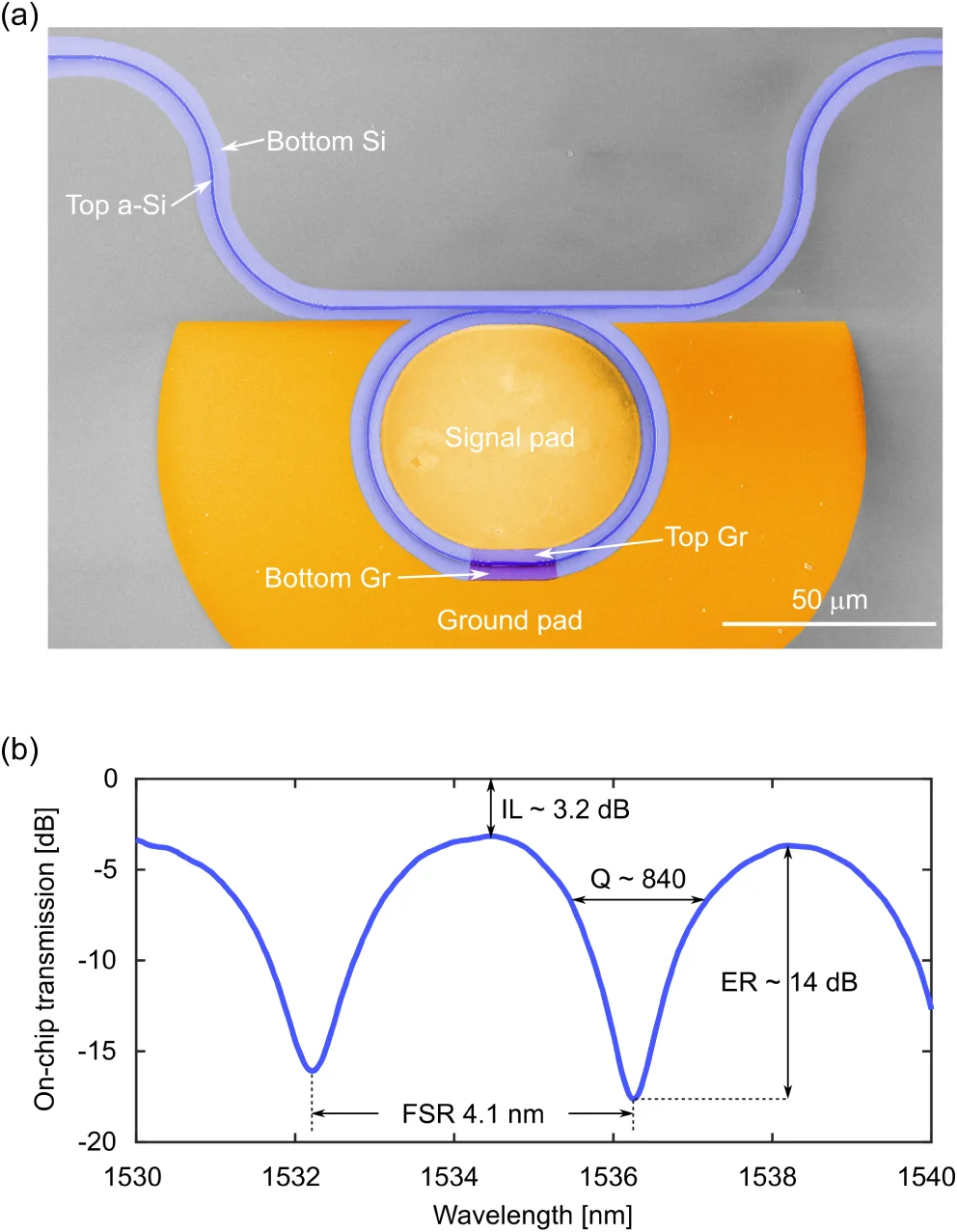
(a) False-color SEM image of a fabricated GOH resonant modulator. Laser beam is coupled in and out of the bus waveguide via grating couplers (GCs). Optical power is fed into the racetrack resonator through a directional coupler. In the active section of the device, bottom and top graphene electrodes are inserted, sandwiching the NLO and connecting to the metal contact pads through graphene edge contact. (b) On-chip transmission of the characterized device. The on-state insertion loss is ∼3.2 dB. The resonances in the C-band feature a moderate *Q*-factor of ∼840 and a large extinction ratio of ∼14 dB.

In this work, four-layer chemical vapor deposited (CVD) graphene sheets were transferred to the fabricated chip for both bottom and top electrodes. Compared to exfoliated graphene flakes, CVD graphene sheets are suitable for chip- or wafer-scale fabrication, but they typically exhibit higher defect densities and smaller domain sizes. In addition, the transfer and especially the patterning process introduce unwanted defects to the graphene electrodes. As a result, their higher scattering rate of carriers leads to higher optical losses than those predicted by simulations. From cut-back measurements, the active phase shifters with 2× four-layer graphene electrodes feature an average propagation loss of 0.093 dB/μm.^30^ The propagation loss of the active phase shifter can be further reduced by using high quality graphene films and a better controlled transfer technique.

Among the fabricated devices, we present a resonant modulator embedded with a 50 μm long active phase shifter. The measured on-chip transmission spectrum for the resonant modulator is plotted in Figure 3(b). The on-state insertion loss was measured to be ∼3.2 dB ± 2.2 dB for a 90% nominal coverage. The measurement uncertainty comes from the nonuniform fabrication quality of the grating couplers. From the breakdown of the optical losses (Supporting Information 4), we clearly see that the insertion loss can be reduced by improvement of the a-Si quality, hence the reduction in the total roundtrip losses. *Q*-factors of the resonances in the C-band were measured to be ∼840, featuring a moderately sharp resonance for a balanced modulation enhancement and a high photon-lifetime limited bandwidth above 200 GHz. The resonances also show a large extinction ratio (ER) of ∼14 dB.

To measure the electro-optic modulation performance of the device, the NLO material was first functionalized by applying an electric field under an elevated temperature. The transfer function of the resonant modulator is plotted in Figure 4(a). Full switching with a 14 dB on–off ratio can be achieved with a voltage of 13 V which shifts the roundtrip optical phase by π. Therefore, the corresponding modulation efficiency for the 50 μm long integrated phase shifter is *V*_π_ *L* = 0.65 V·mm, indicating a Pockels coefficient of the NLO layer of *r*_33_ = 70 pm/V which is twice as large as the one of LiNbO_3_. Note that in data transmission, full on–off is commonly not necessary. Thanks to the resonance enhancement, the signal voltage can be significantly reduced while keeping a high ER and signal-to-noise ratio (SNR). While operating at the 3-dB point, an ER of 6 dB can be achieved with a driving voltage of 2.9 V_p_.

**Figure 4:**
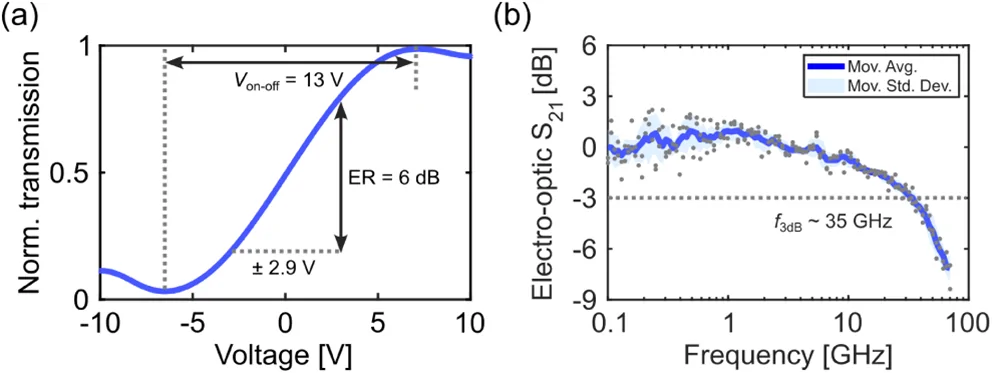
Electro-optic characterization of the resonant modulator. (a) Transfer function measured with 100 kHz triangular wave. An on–off voltage of 13 V was measured with an on–off ratio above 14 dB. Operating at the 3-dB operation point, an extinction ratio of 6 dB can be achieved with a signal voltage of 2.9 V_p_. (b) Frequency response from 0.1 to 70 GHz. It shows a 3-dB bandwidth of ∼35 GHz, and a 6-dB bandwidth of ∼57 GHz. The solid line and shaded area represent the moving average and moving standard deviation of the measured data points.

Subsequently, the frequency response of the fabricated device was tested. Sinusoidal RF signals from 100 MHz to 70 GHz were generated by an RF source to drive the resonant modulator. The modulated output was characterized by an optical spectrum analyzer (OSA). No DC bias was applied during measurements. As shown in Figure 4(b), the device shows a 3-dB bandwidth of ∼35 GHz and a 6-dB bandwidth ∼57 GHz. The limitation in bandwidth mainly comes from the imperfection of graphene resistance. The transferred CVD graphene layers tend to possess more defects and hence, lower carrier mobility than the mechanically exfoliated crystalline graphene flakes.^41^ Especially on top of the NLO layer, electrical measurements revealed an average sheet resistance of 2099 Ω/□ for the top graphene, which is more than 7 times as high as the one from the bottom graphene electrode (∼285 Ω/□).^30^ By further improving the fabrication technology, the 3-dB bandwidth of the GOH resonant modulators can be increased beyond 90 GHz by reducing the resistance of the top graphene electrode to match that of the bottom graphene electrode, as shown in Supporting Information 5.

### High-Speed Data Transmission

High-speed data transmission performance of the activated resonant modulator with 50 μm long graphene electrodes was tested in the back-to-back IM/DD system shown in Figure 5(a). Jointly benefiting from the high modulation efficiency, the large bandwidth and the resonance enhancement, the GOH resonant modulator can transmit high data rates under low driving voltages using computation-friendly digital signal processing (DSP). An arbitrary wave generator (AWG) was used to generate electrical signals and drive the modulator under test with a maximum peak voltage of 1.35 V_p_. The modulated optical output was sent to the preamplified direct detection receiver. Offline linear DSP was performed on the received signals to characterize the transmitted signal quality (see Methods).

**Figure 5:**
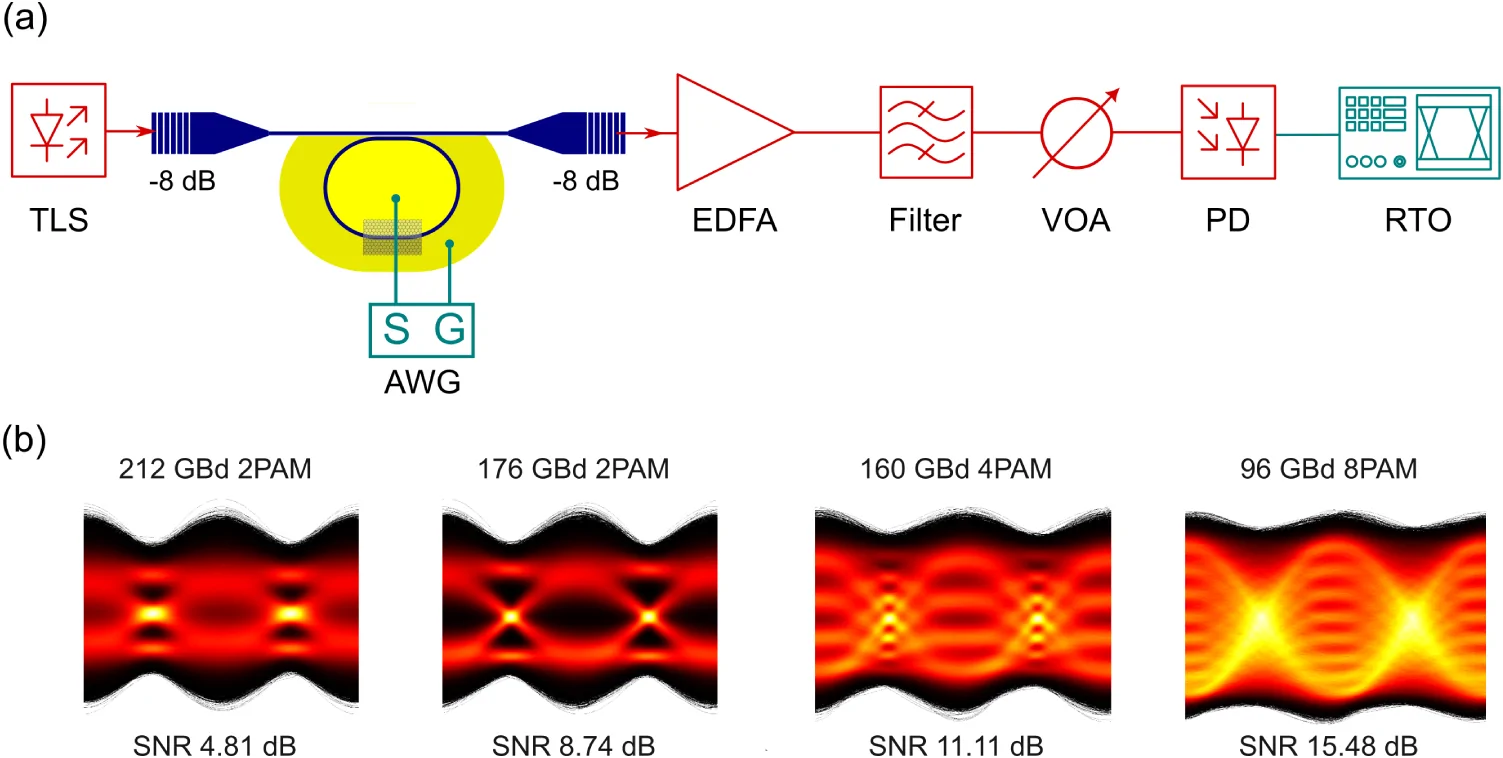
High-speed data measurement with the fabricated GOH resonant modulator. (a) Data measurement setup. 2PAM, 4PAM and 8PAM signals with voltages up to 1.35 V_p_ were sent to the GOH resonant modulator to modulate the C-band carrier generated by TLS. The modulated output signals were received with a preamplified direct detection receiver. (b) Eye diagrams of transmitted 212 GBd 2PAM, 176 GBd 2PAM, 160 GBd 4PAM and 96 GBd 8PAM signals under full voltage swing of *V*_p_ = 1.35 V after timing recovery and least mean square (LMS) equalization.

Under the full voltage swing of 1.35 V_p_, the 212 GBd 2PAM, 176 GBd 2PAM, 160 GBd 4PAM and 96 GBd 8PAM signals have been successfully transmitted. The eye diagrams after applying linear DSP are depicted in Figure 5(b). For the 212 GBd 2PAM signal, we achieved an SNR of 4.81 dB with a normalized generalized mutual information (NGMI) of 0.8554 that is above the 20%-overhead soft-decision forward error correction (SD-FEC) limit of 0.8512.^42,43^ The 176 GBd 2PAM signal was received with an SNR of 8.74 dB and a bit-error-ratio (BER) of 2.82 × 10^–3^ which is below the 6%-overhead hard-decision forward error correction (HD-FEC) limit of 4.7 × 10^–3^.^43^ The 160 GBd 4PAM signal and 96 GBd 8PAM signals were transmitted successfully with NGMIs of 0.8566 and 0.8297 respectively, both of which above the 25%-overhead SD-FEC limit of 0.8116. The offline DSP included a timing recovery and a *T*/2-spaced least mean square (LMS) equalization with 151, 21, 49, and 151 taps for the eye diagrams above, respectively. Applying the linear DSP above with low-to-moderate complexity is enough for achieving high line-rate results. So, for instance, the 160 GBd 4PAM signal with the 49-tap LMS equalization results in a 320 Gbit/s line rate—to the best of our knowledge the highest line rate for an electro-optic modulator using graphene electrodes.

Yet, nonlinearities can still be observed from the imbalance of signal traces. This can be solved by an additional nonlinear DSP step. Applying an additional maximum-a-posteriori (MAP) equalizer of length 3 and a second LMS filter with 1001 taps results in a further SNR and NGMI improvement, see Supporting Information 6.

The energy consumption of the device is appealingly low particularly when the device is operated with low driving voltages. Toward this end, we studied the modulation performance for a 128 GBd 2PAM signal under various driving voltages, see Figure 6. Under driving voltages of 0.85 V_p_ and 0.6 V_p_, we measured BERs of 1.31 × 10^–4^ and 2.72 × 10^–3^ respectively, both of which are below the 5%-overhead HD-FEC limit. When the driving voltage was further reduced to 0.4 V_p_, the transmitted signal showed an SNR of 5.21 dB and an NGMI of 0.874, which is still above the 20%-overhead SD-FEC threshold.

**Figure 6:**
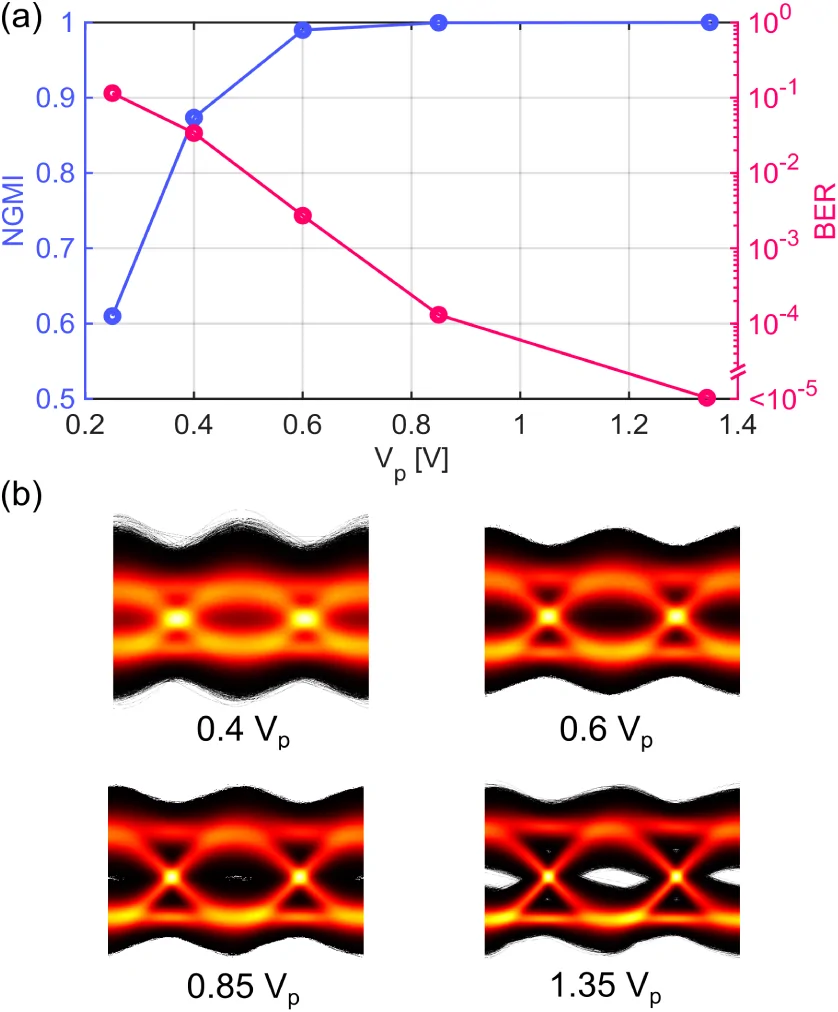
Data measurement results for 128 GBd 2PAM signal under different driving voltages. (a) Normalized general mutual information (NGMI) and bit error rate (BER) as a function of the driving voltage. (b) Eye diagrams of the transmitted signals after timing recovery and LMS equalization with only 9 taps. The transmission reaches below SD-FEC limit at 0.4 V_p_ and below HD-FEC limit above 0.6 V_p_.

To estimate the energy consumption for operation at the low driving voltage of 0.4 V_p_, we performed the following calculation. At a driving voltage of 0.4 V_p_, the on-device peak voltage is doubled to 0.8 V_p_ due to the nearly capacitive load.^44^ Assuming a transition voltage of *V*_pp_ = 2*V*_p_ and equal possibilities of ones and zeros, one may take into account the device RC-limited bandwidth and estimate the energy consumption per bit by,^35,45^

$$E_{\mathrm{bit}}=\frac{1}{2}C_{\mathrm{PS}}V_{\mathrm{pp}}^{2}\left[1-e^{-\frac{T}{\tau }}\right]\cdot \frac{1}{2}=C_{\mathrm{PS}}V_{p}^{2}\left[1-e^{-\frac{T}{\tau }}\right]$$

Here, *C*_PS_ is the phase shifter capacitance, *V*_P_ = 0.8 V is the on-device peak voltage, *T* = 1/128 ns is the symbol duration for the 128 GBd signal, and τ = 1/(2π*f*_RC_) = 1/70π ns is the RC-constant. Using *C*_PS_ ≈ 29.3 fF obtained from the electrostatic simulation, the estimated energy consumption is ∼15.4 fJ/bit. To validate this estimation, we performed electrical S_11_ measurement on the prototyping device (see Supporting Information 7), and we measured the device capacitance to be *C*_PS_ ≈ 19.2 fF. With the measured value, the energy consumption is calculated as ∼10.1 fJ/bit.

The low energy consumption in combination with the low DSP complexity is particularly noteworthy for the case of the 128 GBd 2PAM signal. The signal requires a timing recovery and a *T*/2-spaced LMS equalization with only 9 taps, resulting in rather low computational demands and latency. This shows the capability of the GOH resonant modulator for >100 Gbit/s high-speed data transmission under CMOS-compatible voltages with femtojoule energy consumption, enabled together by the high modulation efficiency, the large bandwidth, and the resonance enhanced modulation depth.

## Discussion and Conclusion

With the demonstration of high-speed energy-efficient data transmission, we have shown the great potential of graphene in optical communication links. Instead of relying on the nonlinearities of graphene itself, integration with other highly nonlinear materials can further enhance device performance.

On our GOH platform, the high-speed performance of the modulator is strongly determined by the resistance of graphene electrodes. The quality of graphene, transfer method and patterning techniques of graphene electrodes are critical to minimize the sheet and contact resistances. Reducing the resistance of the top graphene electrodes to the level of the bottom graphene can boost the modulation bandwidth above 90 GHz. Additionally, proper encapsulation of the graphene electrodes can be introduced to improve their carrier mobility and electrical conductivity. For example, hexagonal boron nitride (hBN) as an encapsulation layer has been proven to reduce the sheet resistance of graphene to ∼40 Ω/□.^46^ With this value, the bandwidth of the resonant modulator can be further increased to above 100 GHz. A further reduction in insertion loss is also expected with a better graphene quality after the transfer and patterning process. Currently, the compromised graphene quality on top of the NLO layer could potentially contribute to variations in device performance. Further systematic optimization of the fabrication techniques is therefore expected to improve process control and device-to-device reproducibility. The poling process for the functional NLO material can still be optimized to reach a higher Pockels coefficient up to 300 pm/V,^20^ which leads to a 4-time higher modulation efficiency compared to the prototyping device. Thermal stability and long-term reliability of the devices heavily depend on the glass-transition temperature of the NLO material. Modulation performance typically degrades when the operating temperature is close to the glass temperature due to the breaking of chromophore alignment. Currently, we estimate that our GOH resonant modulator similarly degrades at operating temperatures ∼85 °C, which is close to the glass-transition temperature of JRD1. By using NLO materials with high glass-transition temperature >170 °C, thermal stability of a plasmonic resonant modulator has been demonstrated.^23^ Alternatively, long-term stability can be substantially improved by replacing the NLO material to a cross-linkable variant,^47^ which has proven to work reliably for 2000 h under 85 °C. The integration of other materials with strong nonlinearities, such as barium titanate (BTO),^48^ onto the versatile platform is also feasible for realizing high-speed, low-loss, and energy efficient modulator devices.

In summary, we have proposed and demonstrated a graphene-organic hybrid resonant modulator on the silicon-on-insulator platform. The employed graphene electrodes not only offer a great electrical conductivity that supports a high RC-limited 3-dB bandwidth (∼35 GHz) but also lower the optical losses together with the carefully designed TM-polarized waveguide. The 50 μm long low-loss active phase shifter features a high modulation efficiency of *V*_π_ *L* = 0.65 V·mm, and is embedded in a racetrack resonator with a compact footprint of 65 μm × 75 μm. Enhanced by the optical resonance, the resonant modulator supports data modulation up to 320 Gbit/s, and achieves 128 Gbit/s operation under CMOS-compatible voltages with femtojoule energy consumption per bit, competing with state-of-the-art silicon electro-optic modulators. Further improvements in graphene quality and fabrication techniques can lower the insertion loss and boost the modulation bandwidth to above 100 GHz, showing the great potential of the graphene integration into high-performance electro-optic devices for optical communication applications.

## Methods

### Fabrication

The prototyping devices were fabricated on a silicon-on-insulator wafer with a 110 nm thick silicon and 3 μm thick buried oxide. The buried silicon slabs were first patterned and structured with local oxidation of silicon (LOCOS) technology.^49^ A layer of dielectrics was deposited by atomic-layer deposition (Oxford FlexAL) for electrical isolation. Bottom contact pads (Ti/Au) were structured by liftoff, and a four-layer CVD graphene sheet was transferred on top by standard wet transfer method. The graphene electrodes were patterned by e-beam lithography (Vistec EBPG 5200+) and structured by reactive-ion etching (Oxford PlasmaPro NPG 80). Edge contact on bottom graphene was formed by a following liftoff step of Cr/Pd/Au. A thin layer of NLO material (JRD1) was spin-coated onto the chip subsequently. A layer of silicon dioxide was deposited on the NLO layer by e-beam evaporation (Plassys MEB550SL3) as the protection layer. Top contact pads were patterned by e-beam lithography and structured by selective wet etching. Next, a second four-layer CVD graphene sheet was transferred, patterned and etched by reactive-ion etching to form the top graphene electrodes. Currently, the top graphene is dry transferred with a PDMS stamping technique. To protect the underlying NLO material, organic solvents are avoided during the process of graphene transfer and patterning. These processing constraints could be an issue that resulted in a much lower graphene quality compared to the standard wet transferred bottom graphene. A feasible solution is to transfer and pattern the graphene electrodes on a solid substrate together with the edge contacts, then transfer the fabricated structures on top of the NLO layer with microtransfer printing technique.^50,51^

Subsequently, a thin layer of alumina was deposited by atomic-layer deposition (Picosun Sunale R-150). Lastly, top a-Si layer was deposited by plasma-enhanced chemical vapor deposition (Oxford PlasmaPro 100 PECVD), patterned by e-beam lithography and structured with inductively coupled plasma reactive-ion etching (Oxford PlasmaLab 100) to finalize the device.

### Passive Optical Characterization and Graphene Resistance Measurement

In passive optical measurements of the device, continuous-wave laser in C-band generated by a tunable laser source (TLS, Keysight 81600B) was coupled into the device via a GC which had a coupling loss of ∼8 dB. The optical output of the device was coupled via a second GC into the optical power meter (Keysight 81364B). The transmission spectrum was measured by sweeping the laser wavelengths. The on-chip transmission was obtained by de-embedding the fiber-to-chip coupling losses from the input and output GCs, which were measured in a reference waveguide. The on-chip transmission uncertainties were characterized by analyzing the transmission spectra of multiple sets of identical reference waveguides.

The resistances of the graphene electrodes were measured with four-contact van der Pauw structures below and above the NLO layer. The voltage–current measurements were performed with a probe station (Cascade SUMMIT200) and Keysight B1500A Semiconductor Device Parameter Analyzer. A DC current is injected through two adjacent contacts, and the voltage drop is measured across the remaining contacts. The extracted average sheet resistance and contact resistivity were 285 Ω/□ and 229 Ω·μm for the bottom graphene electrodes, respectively, compared with 2099 Ω/□ and 1160 Ω·μm for the top graphene electrodes.

### Device Activation and Active Performance Characterization

After the passive characterization, the device was activated by applying a voltage across the NLO layer via the graphene electrodes under an elevated temperature above the glass-transition temperature of JRD1. The activation process was to align the NLO chromophores to break the centrosymmetry and induce the macroscopic electro-optic Pockels effect.

The transfer function was obtained from a low frequency modulation measurement.^18,52^ A low-frequency (*f* = 100 kHz) triangular electrical signal generated by an AWG (Agilent 33500B) with peak voltage *V*_d_ was applied to drive the modulator. Continuous-wave laser from the TLS was coupled into the modulator and the modulated output signal was fed into a photodetector (Koheron PD01). The electrical output of the photodetector was monitored by an oscilloscope. When 2*V*_d_ > *V*_on–off_, overmodulation was achieved. From the waveform between fully on and off states, the transfer function was extracted.

To measure the frequency response, sinusoidal RF waves from 100 MHz to 70 GHz were generated by an RF signal generator (Agilent E8257D) and fed to the modulator through an RF cable and a high-speed RF probe. The TLS wavelength was set to the operation point, and the modulated output was directly fed into an OSA (APEX AP2683B). The intensities of the first-order modulation sidebands were recorded for all frequency points. After de-embedding the RF losses from the RF cable and the RF probe, the electro-optic 3-dB bandwidth ∼35 GHz of the device was obtained.

### High-Speed Data Transmission Experiment

The high-speed data measurement setup for the GOH resonant modulator is shown in Figure 5(a). We used a TLS to generate a continuous-wave laser carrier in the C-band and coupled it into the device via a GC. The laser power was set to 12 dBm, and the coupling loss of the input GC is about 8 dB, which led to an on-chip input optical power of roughly 4 dBm. A high-speed AWG (Keysight M8199B) was used to generate the periodically repeated sequence of at least 2^19^ bits. The modulator was driven by the generated data signals through an RF cable and a high-speed RF probe with total RF losses around 2 dB at 67 GHz. The modulated optical signal was coupled out via a second GC, amplified by an erbium-doped fiber amplifier (EDFA) to 20 dBm and filtered by a 2 nm optical bandpass filter. Subsequently, the power was set using a variable optical attenuator (VOA) and received by a 145 GHz high-speed photodetector. The generated electrical signal was sampled by a 256 GSa/s real-time oscilloscope (RTO, Keysight UXR1104A). The single-ended driving voltage for 212 GBd 2PAM, 176 GBd 2PAM, 160 GBd 4PAM and 96 GBd 8PAM signals was 1.35 V_p_. For the 128 GBd 2PAM signal, reduced driving voltages of 0.85 V_p_, 0.6 V_p_, and 0.4 V_p_ were applied to demonstrate the CMOS-voltage compatibility and energy efficiency. All the reported voltage values are with respect to 50 Ω load excluding the RF losses from the used cable and probe. Below the bandwidth limit, the on-device driving voltage is expected to double due to the capacitive load under the lumped-element assumption, which was taken into account for the calculation of energy consumption of the modulator.

Offline linear DSP was performed on the recorded signals including a timing recovery and a *T*/2-spaced LMS equalization. For the reported 212 GBd 2PAM, 176 GBd 2PAM, 160 GBd 4PAM and 96 GBd 8PAM signals, LMS with 151, 21, 49, and 151 taps were applied, respectively. For the 128 GBd 2PAM signal, LMS with only 9 taps was used for lowest latency and minimal additional energy consumption from the digital electronics. Additional DSP steps consisting of a maximum-a-posteriori (MAP) equalizer of length 3 and a second LMS filter with 1001 taps were performed on the 212 GBd 2PAM, 160 GBd 4PAM and 96 GBd 8PAM signals to further improve their signal quality (see Supporting Information 6).

## Supplementary Material



## Notes

Simulation of the GOH phase shifter, simulated modulation performance of the GOH resonant modulator, OMA enhancement from the racetrack resonator, passive resonator characterization and optical loss breakdown, achievable RC-limited bandwidth with improved top graphene, additional results in high-speed data transmission, equivalent circuit and electrical S_11_ measurements, and comparison of electro-optic modulators (PDF)

## ∇

NLM Photonics, Seattle, WA 98195, USA
