# Supplementary material for: High-Speed Energy-Efficient Resonant Modulator on a Graphene-Organic Hybrid Platform
Source: ACS Photonics. 2026 Oct 24;13(21):6339–47. doi: 10.1021/acsphotonics.6c00793 (PMC13742289; doi:10.1021/acsphotonics.6c00793)
Supplement: Supplementary file 1 [file ph6c00793_si_001.pdf]

Supporting Information for

High-speed Energy Efficient Resonant Modulator on a Graphene-Organic Hybrid Platform

Xinzhi Zhang[1,], Ping Ma[1,2,*], Tobias Blatter[1], Yannik Horst[1], Wolfgang Heni[3], Alexandros Emboras[4], Tatsuhiko Watanabe[1], Nikolaus Flöry[5], Lukas Novotny[5], Delwin L. Elder[6,7], Larry R. Dalton[6], Juerg Leuthold[1,*]*

[1] ETH Zurich, Institute of Electromagnetic Fields (IEF), 8092 Zurich, Switzerland
[2] University of Science and Technology, School of Microelectronics, Hefei, Anhui 230023, P. R. China
[3] Polariton Technologies, 8134 Adliswil, Switzerland
[4] ETH Zurich, Integrated Systems Laboratory, 8092 Zurich, Switzerland
[5] ETH Zurich, Photonics Laboratory, 8093 Zurich, Switzerland
[6] University of Washington, Department of Chemistry, Seattle, WA 98195-1700, USA
[7] Present address: NLM Photonics, Seattle, WA 98195, USA
[*]Corresponding Authors: xinzhi.zhang@ief.ee.ethz.ch, pingma@ustc.edu.cn, Juerg.Leuthold@ief.ee.ethz.ch

Contents:

This Supporting Information contains 8 pages, 8 figures, and 1 table.

Supporting Information 1: Simulation of the GOH phase shifter

Numerical simulations of the active phase shifter were performed using COMSOL Multiphysics with a finite-element method (FEM) mode solver.

For the cross-sectional geometry optimization, we constrained ourselves to 2-D simulations on a layer stack depicted in Figure S1(a). The monolayer graphene electrodes are modeled as thin layers with thickness of $t_G = 0.34$ nm with anisotropic relative permittivity. The in-plane relative permittivity is obtained from ref.[1]

$$\varepsilon_{r,\parallel}(\omega) = \varepsilon_{r,G} + \frac{i\sigma(\omega)}{\omega\varepsilon_0 t_G}$$

where $\varepsilon_{r,G}$ is the out-of-plane relative permittivity as the one from graphite, and $\sigma(\omega)$ is the optical surface conductivity from ref.[2].

The NLO thickness is fixed at 100 nm, and the graphene electrodes overlap with a width of 500 nm. Fermi levels of both bottom and top graphene sheets are fixed at 0.3 eV, which is lower than half of the photon energy at 1550 nm (~0.8 eV); therefore, interband transition is not prohibited in our simulation. Propagation losses and modulation efficiencies over a range of dimensions of the top a-Si strip such as width $w_{\text{a-Si}}$ and thickness $h_{\text{a-Si}}$ have been investigated, as shown in Figure S1(b) and (c).

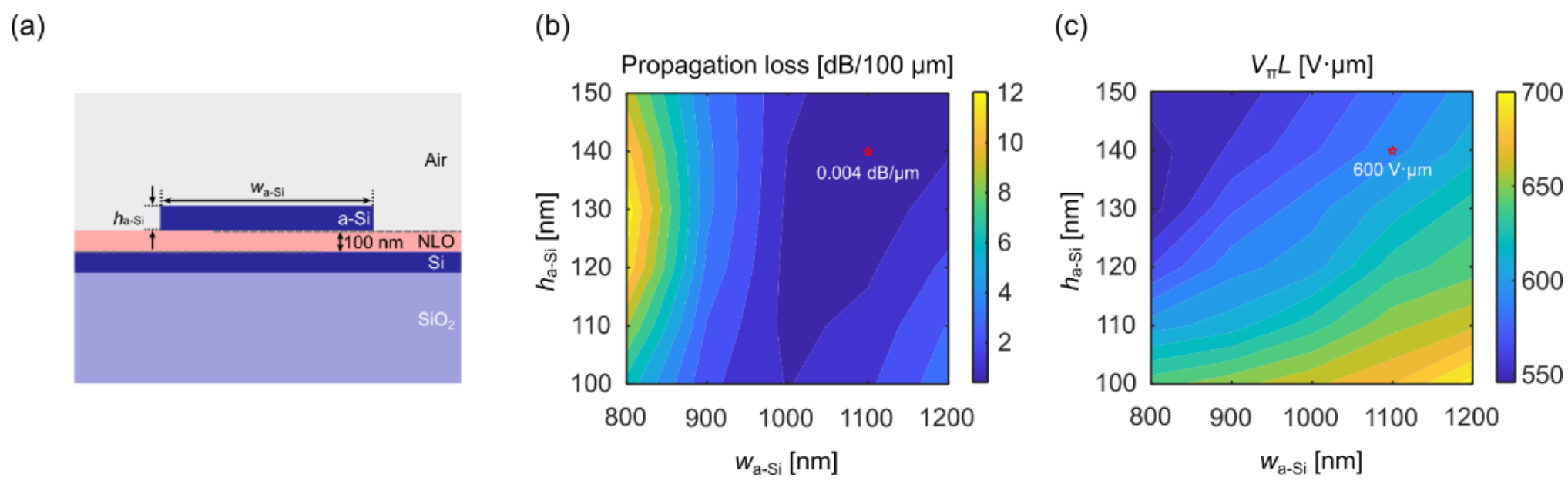

Figure S1. Geometry optimization of the integrated GOH phase shifter. (a) Schematic of the cross-section of the active GOH phase shifter. The NLO thickness is fixed at 100 nm, and the graphene electrodes overlap with a width of 500 nm. (b) Simulated propagation loss with 500 nm graphene overlap as a function of a-Si strip geometry. (c) Simulated $V_\pi L$ of the GOH phase shifter with 500 nm graphene overlap as a function of a-Si strip geometry. Pockels coefficient of the NLO material is presumed to be $r_{33} = 100$ pm/V.

From the simulation results, we picked the dimensions of $w_{\text{a-Si}} = 1100$ nm and $h_{\text{a-Si}} = 140$ nm for the fabricated devices. The propagation loss for the optimized GOH phase shifter is calculated to be ~0.004 dB/µm, corresponding to a $V_\pi L$ of 600 V·µm for a presumed Pockels coefficient $r_{33} = 100$ pm/V.

To extract the Pockels coefficient of the NLO material in the prototyping device, full-wave electromagnetic simulation was performed with the same geometric parameters as the fabricated device. Considering $w_{\text{a-Si}} = 1100$ nm, $h_{\text{a-Si}} = 140$ nm, $t_{\text{OEO}} = 100$ nm and graphene overlap of 1000 nm, the simulated $V_\pi L_{\text{sim}} = 478\ \text{V}\cdot\mu\text{m}$ with a presumed Pockels coefficient $r_{33,\text{sim}} = 100$ pm/V. From the relation between Pockels coefficient and $V_\pi L$

$$V_\pi L = \frac{\lambda_0 d}{n_{\text{OEO}}^3 r_{33} \Gamma}$$

where Γ is the field interaction factor, d is the electrode distance, $V_\pi L$ is inversely proportional to the Pockels coefficient r_{33}.[3] Therefore, we extract the Pockels coefficient to be

$$r_{33,\text{meas}} = \frac{V_\pi L_{\text{sim}} \cdot r_{33,\text{sim}}}{V_\pi L_{\text{meas}}} \approx 73\ \text{pm/V}$$

Supporting Information 2: Simulated modulation performance of the GOH resonant modulator

The active performance of the GOH resonant modulator, such as the OMA and the bandwidth, was simulated in time-domain using MATLAB scripts. The resonant modulator was represented in the simulation by its roundtrip losses, $V_\pi L$, device resistance and capacitance. Initialized with sinusoidal RF signals with a certain driving voltage and various frequencies, the output of the simulated resonant modulator was monitored in time domain, and the simulated OMA and bandwidth were then extracted accordingly.

In the simulations, the sheet resistance of a monolayer graphene was assumed to be 350 Ω/□, which is a typical value for transferred CVD grown graphene. The conductivity of a multilayer graphene sheet was expected to increase

proportionally with the number of layers. The propagation loss of the embedded GOH phase shifter using monolayer graphene was assumed to be 0.004 dB/μm as stated in Supplementary Information 1, and the propagation loss of a multilayer device was proportionally increased likewise. With an assumed $V_{\pi}L$ = 600 V·μm and signal voltage V_p = 1 V, devices with various graphene layers and electrode lengths were simulated.

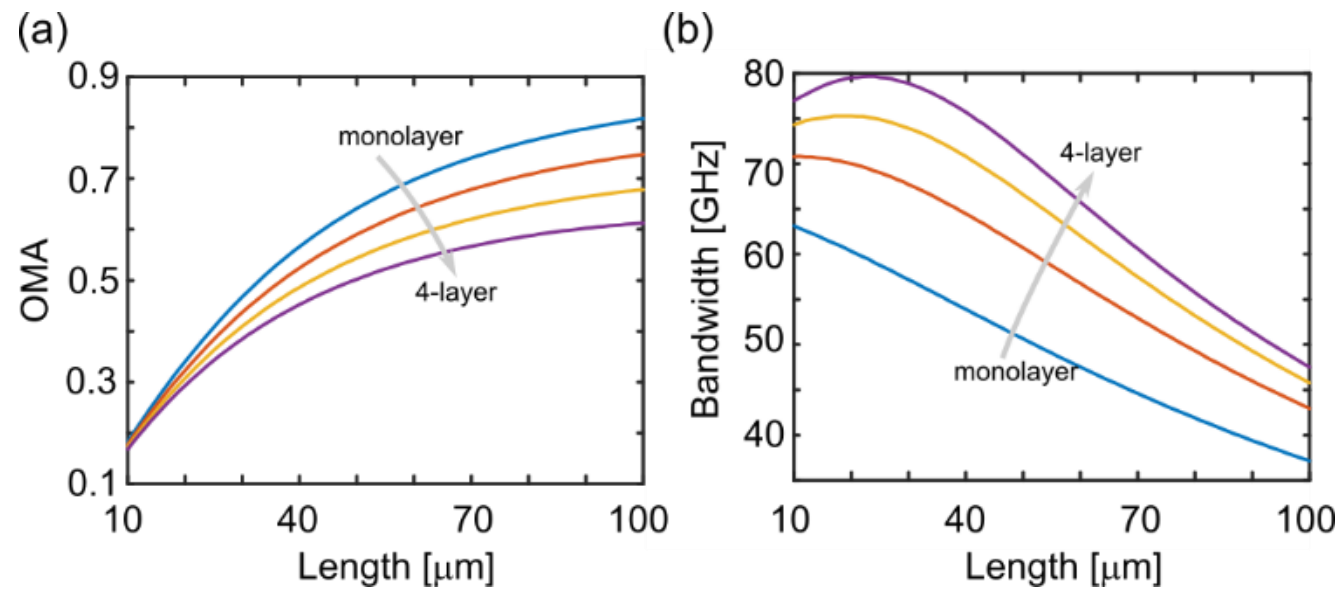

Figure S2. Simulated OMA and RF bandwidth for a GOH resonant modulator under ideal conditions.

From the simulation results, a GOH resonant modulator with monolayer graphene electrodes shows the highest OMA due to the low roundtrip losses and hence a higher Q-factor when the RF frequency is below photon-lifetime limit. In addition, OMA largely benefits from longer active section due to the lower on-off voltage. The simulated bandwidth limitation is a combinational effect of RC-constant and photon-lifetime in cavity. Devices with monolayer graphene suffer from the photon-lifetime limit and the larger graphene resistances and show lower bandwidth compared with multilayer devices. For a modulator with four-layer graphene electrodes, the graphene resistance is minimized, and the adequate roundtrip loss lifts the photon-lifetime limit, therefore, a large bandwidth is predicted. In measurements of the prototyping device, the top graphene electrode shows a much higher resistivity, leading to the reduction in bandwidth. Besides, higher losses from the imperfection in fabrication caused the lower OMA compared to simulation.

Supporting Information 3: OMA enhancement from the racetrack resonator

Modulation performance of the GOH resonant modulators is compared with a GOH MZM in simulation. A MATLAB script was used to calculate the OMA in time-domain. The measured propagation loss of the active phase shifter was applied to calculate the transfer functions of the modulators.

The simulations with a driving voltage of 1V show that the GOH resonant modulator with a 50 μm long phase shifter offers a 67% higher OMA over the OMA of a GOH MZM. This corresponds to an SNR increase of ~4.45 dB in the receiver. In other words, the resonance enhancement can improve the energy efficiency by a factor of ~2.8. To put things in context, it has to be understood that the GOH MZM can be operated in a push-pull operation mode which reduces the on-off voltage by half. Despite this, the resonant GOH modulator has a higher OMA as it not only benefits from the resonant enhancement but also lower overall losses.

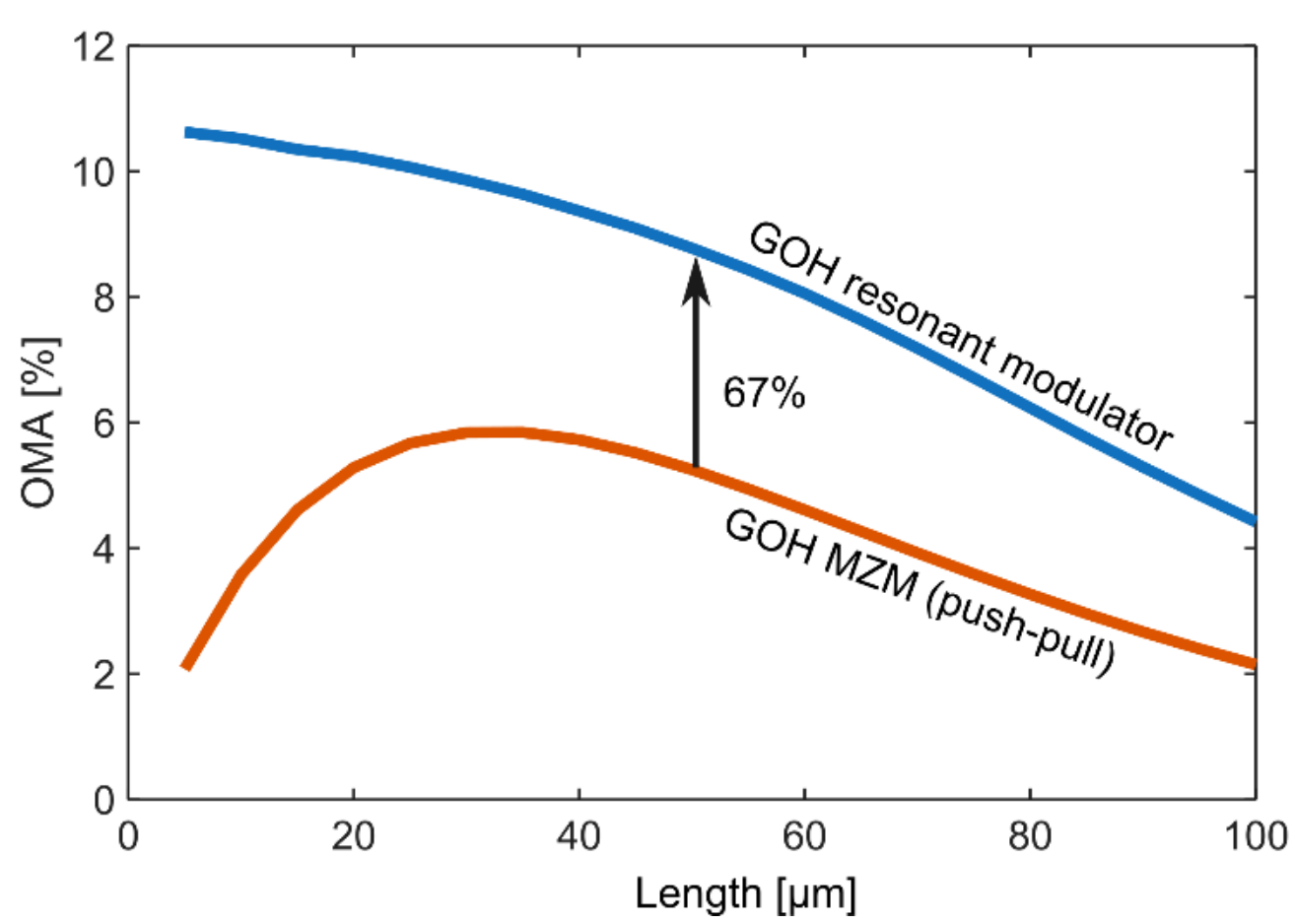

Figure S3. Simulated OMA of the prototyping GOH resonant modulator and GOH MZM at a driving voltage of 1V. When the device length is 50 μm, the resonance enhances the OMA by 67%.

Supporting Information 4: Passive resonator characterization and optical loss breakdown

To explicitly break down the losses of the active resonant modulator, we characterized the passive transmission spectrum of a purely passive racetrack resonator consisting of the Si – NLO – a-Si layer stack without graphene, as shown in Figure S4.

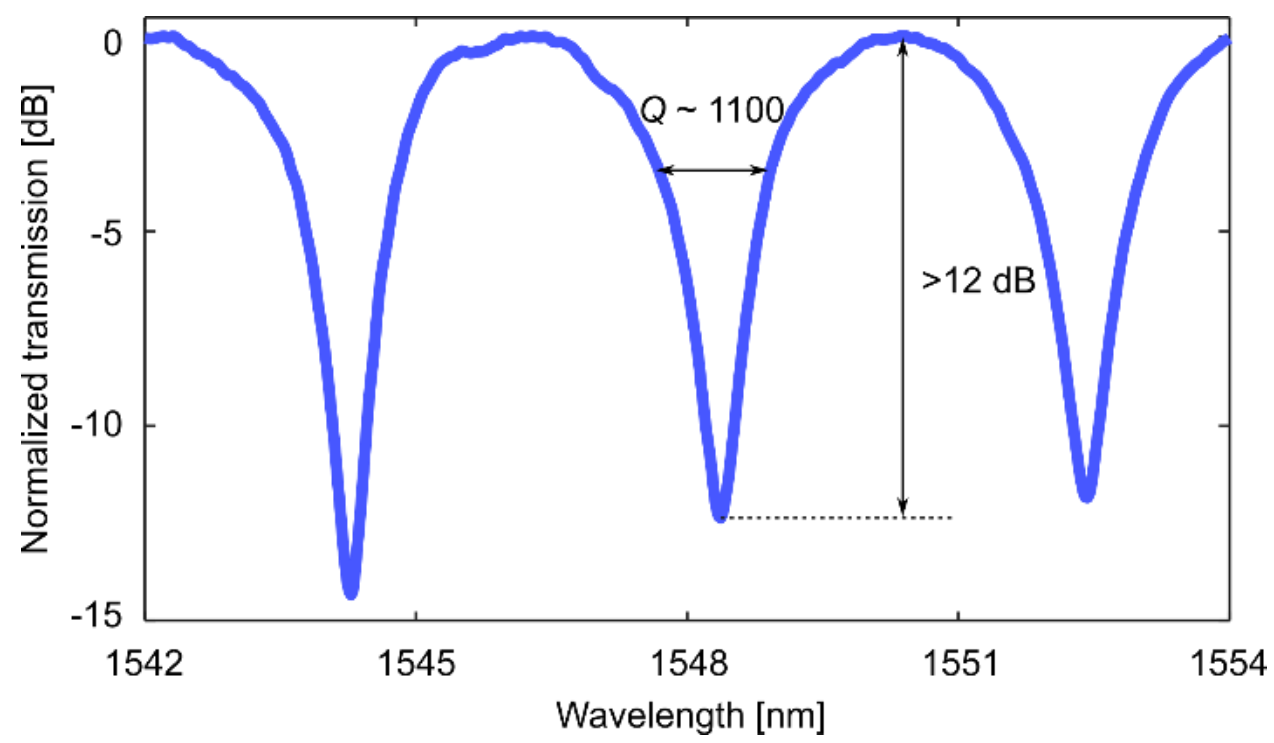

Figure S4. Normalized transmission of a purely passive racetrack resonator without graphene electrodes. Passive roundtrip losses of ~7 dB are extracted.

From the measured Q-factor and extinction ratio, roundtrip bending losses of ~7 dB are extracted.[4,5] The large bending losses originate from the a-Si strip which was deposited and patterned both at low temperatures below 85 °C, leading to fabrication imperfections such as sidewall roughness and geometric variations. By improving the deposition of the a-Si, the bending losses can be largely reduced.[6]

The total roundtrip losses consist of the passive bending losses from the low temperature a-Si ~7 dB and the propagation losses in the 50 µm long active phase shifter ~5 dB. By operating in the under-coupled regime, only a small fraction of optical power is coupled into the racetrack resonator; therefore, the lossy racetrack is bypassed, and the off-resonance insertion loss is much lower than the roundtrip losses.

Supporting Information 5: Achievable RC-limited bandwidth with improved top graphene

Currently, the electro-optic bandwidth of the prototyping GOH resonant modulator is limited by the large resistance of the top graphene. With future improvements in fabrication and a reduction in the top graphene resistance, a higher RC-limited bandwidth could be achieved. Therefore, calculations were performed to estimate the achievable RC-limited bandwidth for various resistances of the top graphene electrodes, as shown in Figure S5. In the calculations, a resonant modulator with a 50 µm long GOH phase shifter is used, assuming a device capacitance $C_\mathrm{d} = 19.2$ fF. Parasitics are not included.

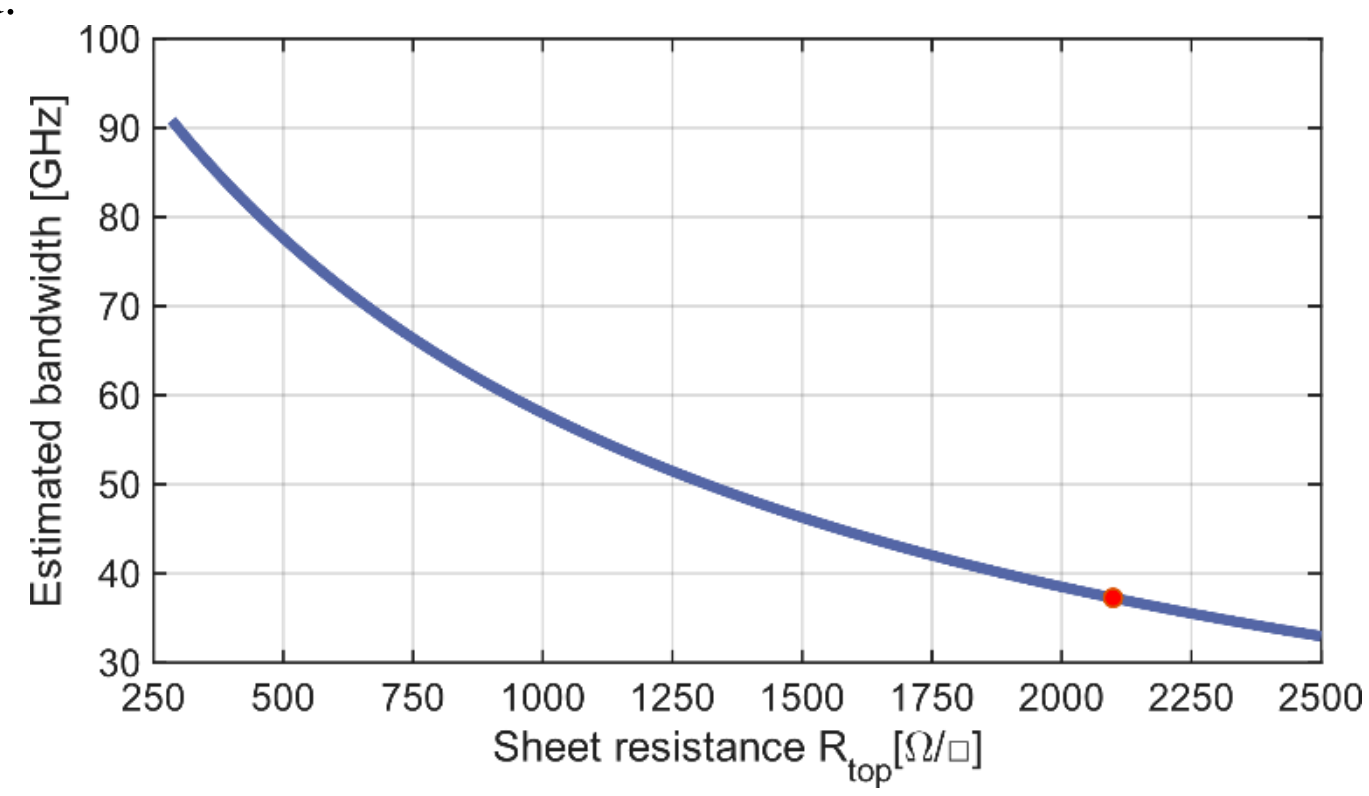

Figure S5. Calculated RC-limited bandwidth for various sheet resistances of top graphene electrodes. The red marker shows the RC-limited bandwidth of the prototyping device.

By improvement of the top graphene quality and transfer method, the estimated RC-limited bandwidth can reach 90 GHz when the bottom and top graphene electrodes have equal resistances.

Supporting Information 6: Additional results in high-speed data transmission

Under full voltage swing of 1.35 V_p, in addition to the results shown in the main text, here we provide the results at lower Baud rates and their corresponding eye diagrams after offline DSP including a timing recovery and an LMS

equalization. For 2PAM, 4PAM and 8PAM signals, we used 151 taps, 49 taps and 151 taps respectively in LMS equalization for consistency with the results shown in the main text.

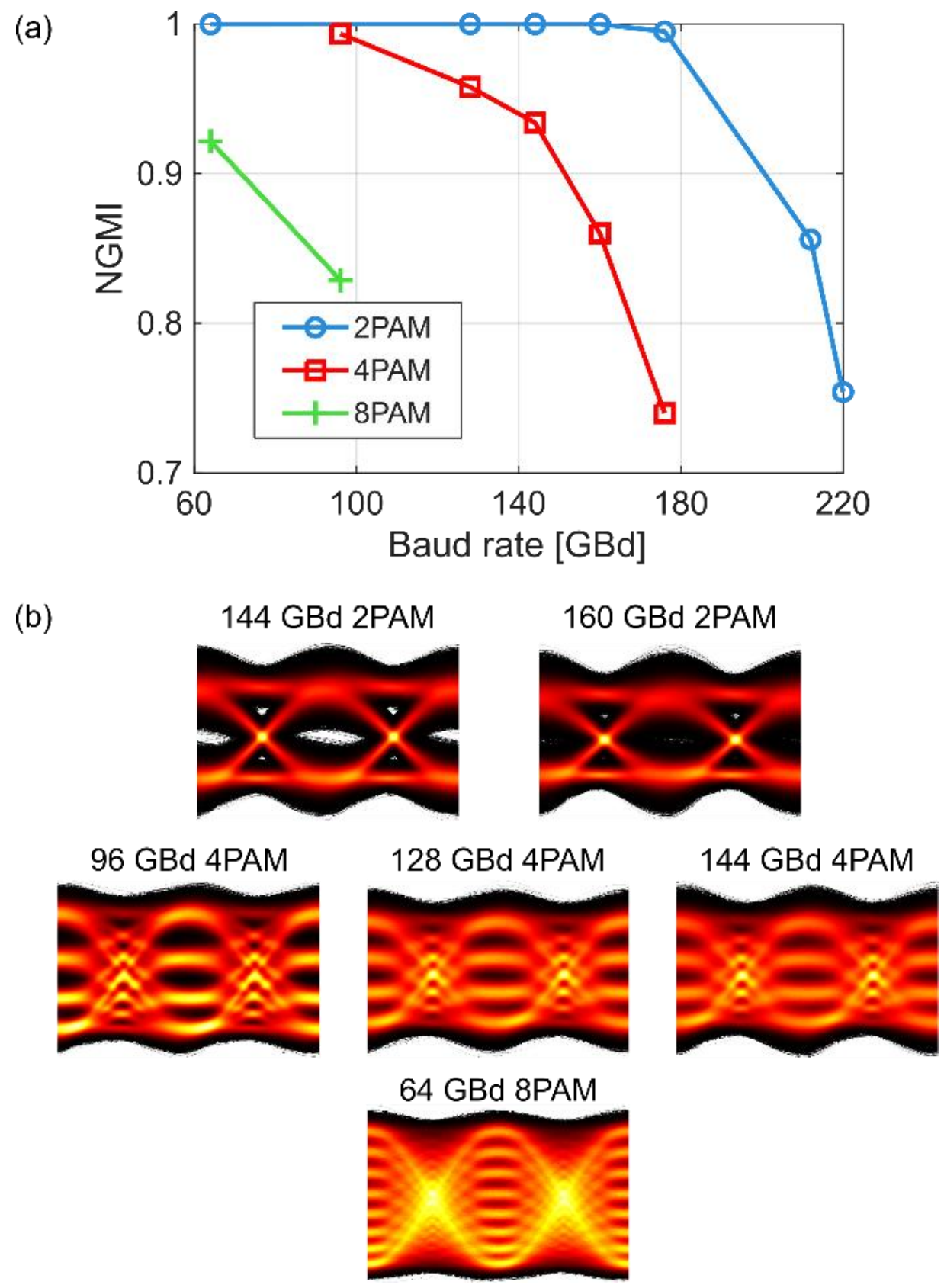

Figure S6. Additional data measurement results. (a) NGMI as a function of Baud rate for different modulation formats. (b) Eye diagrams of transmitted signals after linear DSP.

For ultrahigh Baud rate signals and multilevel modulation formats, even though the device features a resonantly enhanced OMA, simple linear DSP is not sufficient to achieve an optimal signal quality. Therefore, following the linear DSP steps described in Methods, we performed nonlinear DSP including a maximum-a-posteriori (MAP) equalizer of length 3 and a second LMS filter with 1001 taps on the 212 GBd 2PAM, 160 GBd 4PAM and 96 GBd 8PAM signals to further improve the transmission results.

As shown in Figure S7, after applying the additional nonlinear DSP steps, the SNR and NGMI of the received signals were significantly improved, and nonlinearities occurring in the 4PAM and 8PAM signals were substantially reduced. However, nonlinear DSP introduces greater complexity on the receiver side, and a higher latency can be expected.

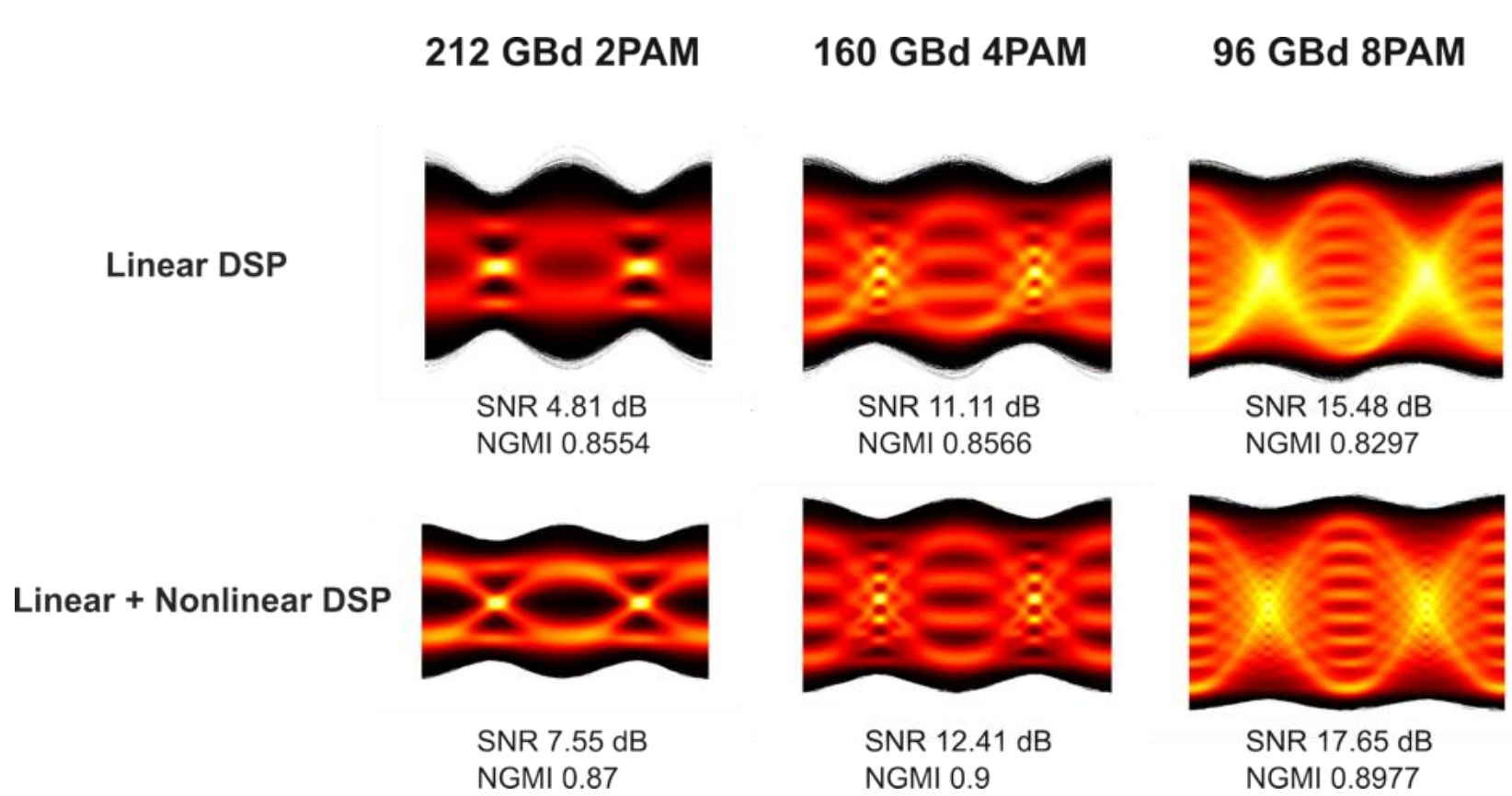

Figure S7. Eye diagrams after linear DSP only and after additional nonlinear DSP. Clear improvement in SNR, NGMI and eye opening can be observed when additional nonlinear DSP is performed.

Supporting Information 7: Equivalent circuit and electrical S_{11} measurements

When not considering the parasitics, the RC-limited bandwidth can be written as

$$f_{\mathrm{RC}} = \frac{1}{2\pi(R_{\mathrm{d}} + R_{\mathrm{s}})C_{\mathrm{d}}}$$

where R_{d} is the device resistance mainly from the graphene electrodes, $R_{\mathrm{s}} = 50\ \Omega$ is the source impedance, C_{d} is the device capacitance from the sandwiched NLO layer. In our measurement, parasitic capacitance exists due to the RF contact pads, therefore, we use the following equivalent circuit model shown in Figure S8(a), where device resistance $R_{\mathrm{d}} = R_{\mathrm{g,bot}} + R_{\mathrm{g,top}}$, C_{p} is the parasitic capacitance.

To experimentally validate the RC-limited bandwidth, we performed electrical S_{11} measurements on the prototyping device from 45 MHz to 50 GHz using a vector network analyzer and 67 GHz RF probes, after calibration with a standard calibration substrate to define the RF measurement reference plane to the probe tip. As shown in Figure S8(b), we fit the measured data using the equivalent circuit model and extracted the parameters as: device capacitance $C_{\mathrm{d}} = 19.2$ fF, parasitic capacitance $C_{\mathrm{p}} = 11$ fF and device resistance $R_{\mathrm{d}} = 172.4\ \Omega$.

Using the extracted results, we can calculate the RC-limited bandwidth as ~ 37.26 GHz excluding the parasitics. The difference between the simulated device capacitance and measured value can come from the lower relative permittivity of the NLO material in high frequency ranges.

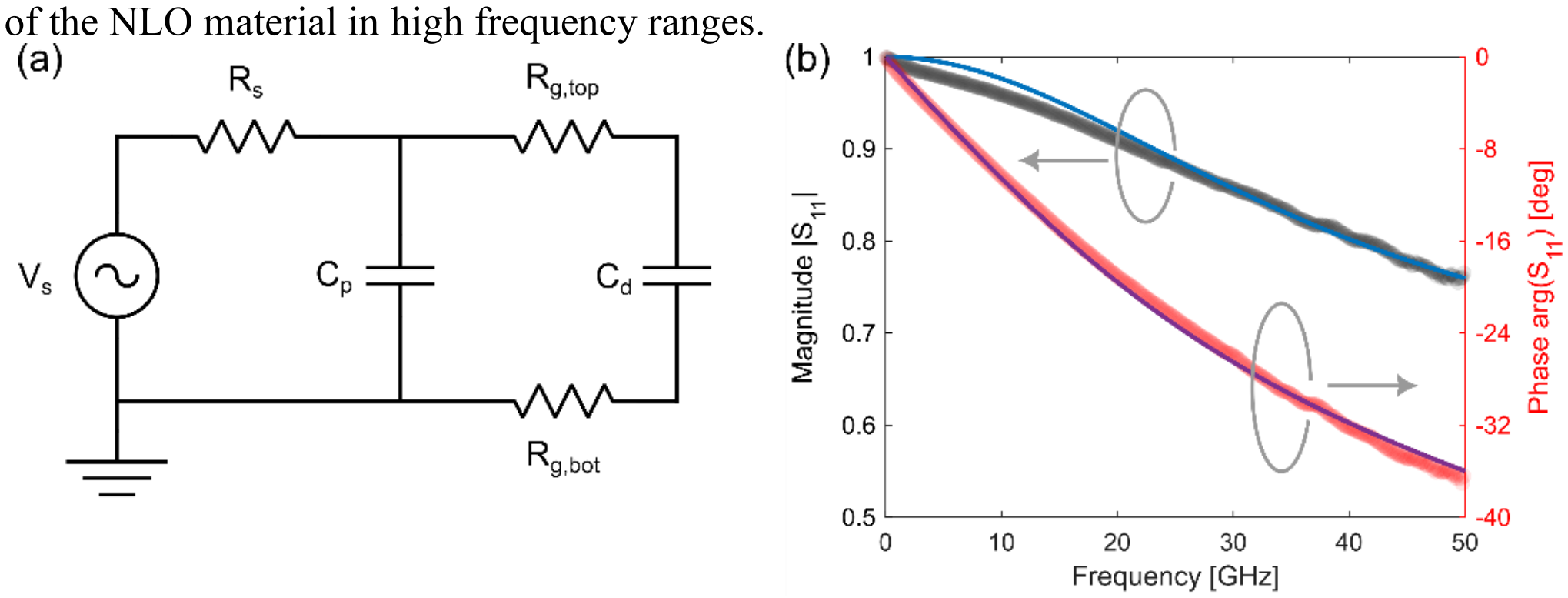

Figure S8. (a) Equivalent circuit for the RF measurements. $R_{\mathrm{s}} = 50\ \Omega$ is the source impedance, $R_{\mathrm{g,bot}}$ is the bottom graphene resistance, $R_{\mathrm{g,top}}$ is the top graphene resistance, C_{d} is the device capacitance and C_{p} is the parasitic capacitance. (b) Magnitude (left axis) and phase (right axis) of the measured S_{11} parameters up to 50 GHz. Fitting the measured data was conducted according to the equivalent circuit.

Supporting Information 8: Comparison of electro-optic modulators

In Table S1, we summarize bandwidth, $V_{\pi}L$, achieved line rate, estimated energy consumption and driving voltage of high-speed electro-optic modulators on various platforms.

Table S1. Literature overview of high-speed energy-efficient electro-optic modulators

Device	Active material	Bandwidth [GHz]	$V_{\pi}L$ [V·µm]	Line rate [Gbit/s]	Energy consumption [fJ/bit]	Driving voltage
**Graphene EAM[7]**	Graphene	67	*	80	58	3.5 V_p + 2 V_{DC}
**Graphene MRM[8]**	Graphene	>70	220	50	~10.5	1 V_p + 5 V_{DC}
**SOH MRM[9]**	Organics	*	477	120	0.0128	0.05 V_p
**POH MRM[10]**	Organics	176	150	408	12.3	0.6 V_p
**Si MRM[11]**	Si	>110	7500	400	0.2	0.5 V_p
**Si MRM[12]**	Si	>110	5700	400	2.89	1 V_p + 3 V_{DC}
**TFLN MRM[13]**	TFLN	>67	3500	240	2.7	0.375 V_p

**TFLN MRM**[14]	TFLN	16	70000	45	*	1.3 V_p
**This work**	Organics	35	650	128	10.1	0.4 V_p
				320	29	1.35 V_p

EAM, electro-absorption modulator. MRM, microring modulator. Values denoted with * are not available.

References

(1) Falkovsky, L. A. Optical Properties of Graphene and IV - VI Semiconductors. *Phys. Usp.* **2008**, *51* (9), 887–897. https://doi.org/10.1070/PU2008v051n09ABEH006625

(2) Hanson, G. W. Dyadic Green's Functions and Guided Surface Waves for a Surface Conductivity Model of Graphene. *J. Appl. Phys.* **2008**, *103* (6), 64302. https://doi.org/10.1063/1.2891452

(3) Chelladurai, D.; Kohli, M.; Winiger, J.; Moor, D.; Messner, A.; Fedoryshyn, Y.; Eleraky, M.; Liu, Y.; Wang, H.; Leuthold, J. Barium Titanate and Lithium Niobate Permittivity and Pockels Coefficients from Megahertz to Sub-Terahertz Frequencies. *Nat. Mater.* **2025**, *24* (6), 868–875. https://doi.org/10.1038/s41563-025-02158-1

(4) Bogaerts, W.; De Heyn, P.; Van Vaerenbergh, T.; De Vos, K.; Kumar Selvaraja, S.; Claes, T.; Dumon, P.; Bienstman, P.; Van Thourhout, D.; Baets, R. Silicon Microring Resonators. *Laser & Photon. Rev.* **2012**, *6* (1), 47–73. https://doi.org/10.1002/lpor.201100017

(5) Blatter, T.; Horst, Y.; Eppenberger, M.; Pappas, C.; Tsakyridis, A.; Giamougiannis, G.; Kohli, M.; Koch, U.; Smajic, J.; Moralis-Pegios, M.; Pleros, N.; Leuthold, J. Ring-Assisted, Racetrack, and Mach-Zehnder Modulator: Which One Offers the Lowest Voltages and Chirp-Free Operation? *Opt. Express* **2024**, *32* (22), 37968–37983. https://doi.org/10.1364/OE.532934

(6) Hsieh, W.-H.; Wu, S.-Y.; Chen, S.-H.; Wang, P.-H. Ultra-Low-Temperature a-Si:H Sputtering for High-Quality Photonic Integration. *Opt. Express* **2026**, *34* (14), 26047–26057. https://doi.org/10.1364/OE.606834

(7) Tiberi, M.; Montanaro, A.; Wen, C.; Zhang, J.; Balci, O.; Shinde, S. M.; Sharma, S.; Meersha, A.; Shekhar, H.; Muench, J. E.; Conran, B. R.; Teo, K. B. K.; Ebert, M.; Yan, X.; Tran, Y.; Banakar, M.; Littlejohns, C.; Reed, G. T.; Romagnoli, M.; Ruocco, A.; Sorianello, V.; Ferrari, A. C. Graphene Electro-Absorption Modulators for Energy-Efficient and High-Speed Optical Transceivers. June 3, 2025. arXiv (Materials Science). arXiv:2506.03281. (accessed 2025-06-10)

(8) Luan, C.; Kong, D.; Liu, Y.; Ding, Y.; Hu, H. High-Efficiency Graphene-Silicon Slot-Waveguide Microring Modulator at 1.5 and 2 µm Wavelength Bands. *Laser Photonics Rev.* **2026**, *20* (19), e71349. https://doi.org/10.1002/lpor.71349

(9) Kholeif, H.; Schwarzenberger, A.; Kotz, A.; Sarwar, S.; Fang, D.; Erk, P.; Bräse, S.; Kuzmin, A.; Freude, W.; Koos, C. Silicon-Organic Hybrid (SOH) Racetrack Modulators for 200 Gbit/s PAM4 Signaling with Ultra-Low Drive Voltages. In *2026 Optical Fiber Communications Conference and Exhibition (OFC)*; IEEE: Los Angeles, CA, USA, 2026; pp 1–3.

(10) Eppenberger, M.; Messner, A.; Bitachon, B. I.; Heni, W.; Blatter, T.; Habegger, P.; Destraz, M.; De Leo, E.; Meier, N.; Del Medico, N.; Hoessbacher, C.; Baeuerle, B.; Leuthold, J. Resonant Plasmonic Micro-Racetrack Modulators with High Bandwidth and High Temperature Tolerance. *Nat. Photonics* **2023**, *17* (4), 361–367. https://doi.org/10.1038/s41566-023-01161-9

(11) Wang, X.; Yang, F.; Wang, X.; Shen, R.; Zhou, Y.; Jia, E.; Wang, A.; Hu, F.; Cai, H.; Chu, W. A 400 Gbps Microring Modulator with 4.2 THz FSR Using Optimized Bezier Bend in a 300 Mm CMOS Platform. *ACS Photonics* **2026**, *13* (14), 3788–3795. https://doi.org/10.1021/acsphotonics.5c02442

(12) Hu, F.; Yu, F.; Liu, X.; Wang, A.; Hu, X.; Cai, H.; Chu, W. 400-Gbps/λ Ultrafast Silicon Microring Modulator for Scalable Optical Compute Interconnects. *Laser Photonics Rev.* **2026**, *20* (13), e02840. https://doi.org/10.1002/lpor.202502840

(13) Xue, Y.; Gan, R.; Chen, K.; Chen, G.; Ruan, Z.; Zhang, J.; Liu, J.; Dai, D.; Guo, C.; Liu, L. Breaking the Bandwidth Limit of a High-Quality-Factor Ring Modulator Based on Thin-Film Lithium Niobate. *Optica* **2022**, *9* (10), 1131–1137. https://doi.org/10.1364/OPTICA.470596

(14) Tan, Y.; Niu, S.; Billet, M.; Singh, N.; Niels, M.; Vanackere, T.; Van Kerrebrouck, J.; Roelkens, G.; Kuyken, B.; Van Thourhout, D. Micro-Transfer Printed Thin Film Lithium Niobate (TFLN)-on-Silicon Ring Modulator. *ACS Photonics* **2024**, *11* (5), 1920–1927. https://doi.org/10.1021/acsphotonics.3c01869
